# Optimized Complex Network Method (OCNM) for Improving Accuracy of Measuring Human Attention in Single-Electrode Neurofeedback System

**DOI:** 10.1155/2019/2167871

**Published:** 2019-03-03

**Authors:** Zheng-Ping Wu, Wei Zhang, Jing Zhao, Chun Chen, Peng Ji

**Affiliations:** ^1^School of Innovations, Sanjiang University, Nanjing 210012, China; ^2^Department of Electrical Engineering, Yanshan University, Qinhuangdao 066004, China

## Abstract

A neurofeedback system adjusting an individual's attention is an effective treatment for attention-deficit/hyperactivity disorder (ADHD). In current studies, an accurate measure of the level of human attention is one of the key issues that arouse much interest. This paper proposes a novel optimized complex network method (OCNM) for measuring an individual's attention level using single-electrode electroencephalography (EEG) signals. A time-delay embedding algorithm was used to reconstruct EEG data epochs into nodes of the OCNM network. Euclidean distances were calculated between each two nodes to decide edges of the network. Three key parameters influencing OCNM, i.e., delaying time, embedding dimension, and connection threshold, were optimized for each individual. The average degree and clustering coefficient of the constructed network were extracted as a feature vector and were classified into two patterns of concentration and relaxation using an LDA classifier. In the offline experiments of six subjects, the classification performance was tested and compared with an attention meter method (AMM) and an *α* + *β* + *δ* + *θ* + *R* method. The experimental results showed that the proposed OCNM achieved the highest accuracy rate (80.67% versus 70.58% and 68.88%). This suggests that the proposed method can potentially be used for EEG-based neurofeedback systems with a single electrode.

## 1. Introduction

A neurofeedback system aiming at building the self-regulation mechanism is commonly used to adjust an individual's brain activity by means of biofeedback. It is an effective treatment for attention-deficit/hyperactivity disorder (ADHD) which is a common disorder in psychiatry with a worldwide prevalence of approximately 5.2% [1]. The major symptom of ADHD is lack of attention, and measuring the human attention is one of the key issues in current researches of the neurofeedback systems. A commonly used technology for measuring the attention level is acquiring electroencephalography (EEG) signals from the electrodes placed on the scalp because of its noninvasive and inexpensive assay, ease of use, and acceptable temporal resolution [2]. Losing attention usually produces changes in the EEG signals of theta (4–8 Hz) and beta (13–20 Hz) bands. Amplitudes of these frequency bands were extracted from an FPz electrode on the forehead to assess the subject's attention level [3]. Although this method is simple in implementation, it is limited to the lack of accuracy. Various methods using data from multiple electrodes, such as relative power spectrum method and independent component analysis (ICA), have also been proposed for improving the performance of measuring attention [4].

However, for the home application and entertainment use, a neurofeedback system with single electrode on the forehead is more widely used because of its inexpensive assay and no need for injecting conductive gel [5, 6]. For such a system decoding single-electrode EEG signals, the major difficulty lies in measuring human's attention accurately. Liu et al. proposed an *α* + *β* + *δ* + *θ* + *R* method, extracting features from multiple wavebands and achieved an accuracy rate of 68.88% in our experiment [7]. NeuroSky Inc., USA, designed an attention meter method (AMM) and implemented it in a ThinkGear ASIC Module (TGAM). The TGAM outputs the AMM values representing individual's attention level and is wildly used in entertainment and educational applications such as MindFlex and Nervanix systems [8]. According to our testing results, the AMM method delivered an average accuracy rate of 70.58%. However, as a nonlinear time series, single-electrode EEG signals are sensitive to noises and artifacts and is difficult to be accurately classified using the frequency-domain features. In this paper, we propose a novel optimized complex network method (OCNM) based on nonlinear time series analysis to measure an individual's attention level. The network is constructed from the single-electrode EEG signals using parameters optimized for each individual, and the average degree and the average clustering coefficient are extracted as features for classification. To validate the effectiveness of the proposed method, we compared its classification accuracy with the AMM and *α* + *β* + *δ* + *θ* + *R* method.

The rest of the paper is organized as follows: Section 2 describes the implementation of the OCNM method and the procedure of the offline experiments. In Section 3, we discuss the experimental results and some issues that arise from our experiments. Lastly, the conclusions and suggestions for future work are given in Section 4.

## 2. Materials and Methods

### 2.1. EEG Data Acquisition

**Figure 1:**
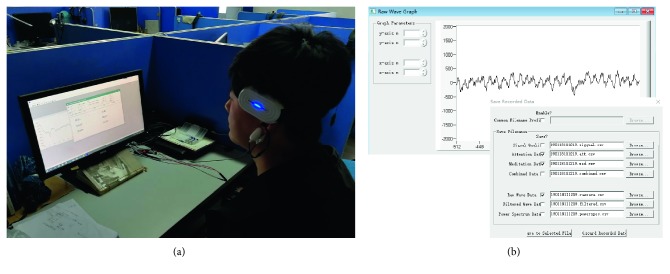
MindFlex headset (a) and recording software (b) for acquiring EEG data.

EEG signals were acquired with a dry-electrode headset (Sichiray Inc., China) designed based on the TGAM. The TGAM is a brainwave sensor module designed by NeuroSky Inc. for sampling and processing the EEG data. It calculated the attention meters using the AMM method and outputted the AMM values along with the raw EEG data. The Sichiray headset uses the TGAM to acquire EEG signals at a sampling rate of 512 Hz with a low signal-to-noise ratio (SNR) and transmits the data including raw EEG and AMM values to a recording system via wireless Bluetooth. The ground and reference electrodes were placed on the subject's left earlobe. The data from a single dry electrode placed at FPz on the forehead were recorded.  Figure 1 shows a picture of the Sichiray headset and data recording during the experiment. In our experiment, recording software was used to save the raw EEG data and the attention data of AMM.

### 2.2. Experimental Procedure

**Figure 2:**
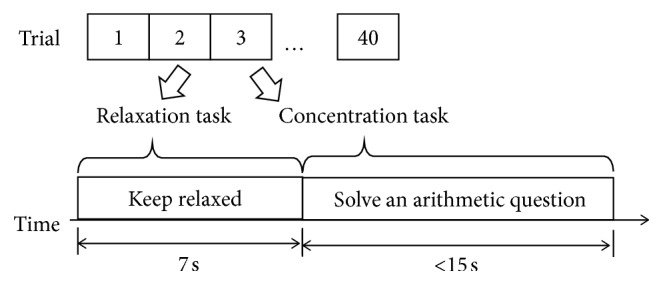
Illustration of the experimental procedure.

The experiments were carried out in a quiet laboratory environment without electromagnetic shielding. Six healthy subjects (1 female, aged 19 to 30 years) participated in this experiment. All subjects were seated in a comfortable armchair and were presented with experimental instructions using an LCD monitor. Each subject underwent a total of 40 trials of experiments, containing two types of mental tasks, i.e., concentration and relaxation. In the concentration task, the subject was instructed to solve a mental arithmetic question, for example, “37 × 89 = ?” [9]. Most of the subjects finished the question in 15 seconds and prepared for the next task of relaxation. In the relaxation task, a blank image was displayed on the LCD monitor and the subject was instructed to keep relaxed. After 7 seconds, the relaxation task was ended and a beep sound for the subjects was played. The experimental procedure is illustrated in Figure 2.

### 2.3. OCNM for Feature Extraction

As EEG signals are a typical nonlinear time series, the nonlinear time series analysis methods including complex network are effective in extracting features of the dynamical EEG signals. To investigate the brainwave patterns from the perspective of network relations, we proposed a novel method for constructing the complex network representing the concentration and relaxation patterns of the single-electrode EEG signals. To establish the network, we used phase space reconstruction (PSR) to quantify the signals from a dynamic perspective [10]. The nodes were reconstructed from a single-electrode EEG signal to characterize its dynamic features in phase space. The EEG data *x*_*i*_, where *i*=1,2,…, *N* is the sampling points of the time series, were reconstructed in an *m*-dimension phase space according to equation (1). The nodes *X* for constructing the network were calculated using the time-delay embedding algorithm for PSR [11]:

(1) $$\begin{array}{c} X=\left(\begin{array}{c} X_{1}\\ X_{2}\\ \cdots \\ X_{M} \end{array}\right)=\left(\begin{array}{cccc} x_{1} & x_{1+\tau } & \cdots & x_{1+\left(m-1\right)\tau }\\ x_{2} & x_{2+\tau } & \cdots & x_{2+\left(m-1\right)\tau }\\ \cdots & \cdots & \cdots & \cdots \\ x_{M} & x_{M+\tau } & \cdots & x_{N} \end{array}\right), \end{array}$$

where *M*=*N* − (*m* − 1)*τ*; *τ* is the delaying time, and *m* is the embedding dimension.

To improve the classification performance, the parameters *τ* and *m* influencing the network structures were optimized separately for each subject. *τ* was optimized using the C-C algorithm based on correlation integral by finding the minima of the following equation [12, 13]:

(2) $$\begin{array}{c} \Delta \bar{S}\left(\tau \right)=\frac{1}{4}\overset{5}{\underset{m=2}{\sum }}\Delta S\left(m,\tau \right)\\ =\frac{1}{4}\overset{5}{\underset{m=2}{\sum }}\left(\max\left\{S\left(m,r_{j},\tau \right)\right\}-\min\left\{S\left(m,r_{j},\tau \right)\right\}\right),\\ S\left(m,r,t\right)=\frac{1}{t}\overset{t}{\underset{s=1}{\sum }}\left\{C\left(m,r,t\right)-C^{m}\left(1,r,t\right)\right\}, \end{array}$$

where *r*_*j*_=*iσ*/2. *C*(*m*, *r*, *t*) is the correlation integral for the embedded time series *X*, and measures the fraction of the pairs of points whose sup-norm separation is no greater than *r*. After acquiring the optimized *τ*, the modified Cao method was adopted to optimize the embedding dimension *m* [14].

The edges regarding the interactions between the nodes were calculated according to Euclidean distances as follows:

(3) $$\begin{array}{c} a_{ij}=\left\{\begin{array}{cc} 1, & \text{if}\;\;d_{ij}\leq \theta ,\\ 0, & \text{if}\;\;d_{ij}>\theta , \end{array}\right. \end{array}$$

where *d*_*ij*_=∑_*n*=1_^*M*^‖*X*_*i*_(*n*) − *X*_*j*_(*n*)‖ is the Euclidean distance between the *i* ^th^ node and the *j* ^th^ node. The threshold *θ* deciding the connectivity between two nodes was chosen for each subject as the minimum value to keep the network fully connected under the concentration state.

To classify the EEG patterns of concentration and relaxation, *K* and the average clustering coefficient *C* were calculated for the constructed network [15]. The average degree *K* reflects the probability that a randomly chosen node has a certain number of links *k*_*i*_ and is calculated as follows:

(4) $$\begin{array}{c} K=\frac{1}{M}\underset{i\in M}{\sum }k_{i}, \end{array}$$

where *k*_*i*_ is the degree of node *i* and *M* is the total number of nodes. *k*_*i*_ is equal to the number of links connected to the node and reflects importance of the individual node in the network [16].

The average clustering coefficient *C* reflects the prevalence of clustered connectivity around individual nodes and is calculated as follows:

(5) $$\begin{array}{c} C=\frac{1}{N}\underset{i\in M}{\sum }C_{i}=\frac{1}{N}\underset{i\in M}{\sum }\frac{\sum_{j,h\in M}{\left(w_{ij}w_{ih}w_{jh}\right)}^{1/3}}{k_{i}\left(k_{i}-1\right)}, \end{array}$$

where *C*_*i*_ is the clustering coefficient for node *i* and *w*_*ij*_ is the weight between nodes *i* and *j*. *C*_*i*_ is equal to the proportion of existing links between the node *i* and its neighbourhood to the maximum possible number of such links. It is a measure of the degree to which nodes in a graph tend to cluster together. The feature vector consisting of *K* and *C* for the network was classified by using a linear discriminant analysis (LDA) classifier to identify the attention level.

### 2.4. LDA Classifier

LDA is a linear classifier aiming at finding a hyperplane to separate the EEG data representing different classes. This paper used LDA to classify the OCNM features because of its low computational requirement and demonstrated classification performance in brain-computer interface (BCI) systems [17, 18]. The use of the LDA classifier contains a training step and a testing step. In the training step, the feature vectors were extracted from the training data of the concentration and relaxation tasks using the OCNM and used to train the LDA classifier to construct a hyperplane to separate two classes of feature vectors. In the testing step, the trained LDA classifier was used to classify the testing data by calculating the distance *d* of its feature vector from the hyperplane:

(6) $$\begin{array}{c} d=w^{\text{T}}p+b, \end{array}$$

where *p* is the feature vector of the testing data. *w* and *b* are the normal vector and the bias parameter representing the hyperplane. The subject's attention level was then categorized as concentration or relaxation according to the sign of the distance.

### 2.5. Performance Evaluation

The EEG data were recorded from FPz electrode during the whole experiment and were divided into 1 sec data epochs between 2 sec and 3 sec in each trial for offline analysis. The data epochs were classified using OCNM into two classes, i.e., concentration and relaxation. The classification performance was evaluated by using the accuracy rate which indicates a percentage of epochs that are successfully classified. In this paper, we calculate the accuracy rate of the OCNM according to 10 × 10-fold cross validation [19]. In each fold, the 40 epochs of recorded data were divided into 36 epochs for training and 4 epochs for testing. The classifier was first trained with 36 training epochs and then was used to classify the 4 testing epochs. The accuracy rate was averaged across all the epochs in 10 × 10 folds.

In the offline experiments, we compared the performance of the OCNM with two methods commonly used in measuring human's attention, i.e., the AMM and the *α* + *β* + *δ* + *θ* + *R* method. As the experiments consisted of two tasks of concentration and relaxation, it is a 2-class classification problem testing different feature extraction algorithms. The AMM developed by NeuroSky Inc. (http://neurosky.com/biosensors/eeg-sensor/algorithms/) is prebuilt in the TGAM to evaluate individual's attention meters. The Sichiray headset is designed based on TGAM and outputs the AMM values ranging from 0 to 100 via Bluetooth. During the experiments, we recorded the AMM values along with the raw EEG data to evaluate performance of the three methods. To calculate the classification accuracy of the AMM, the AMM values recorded in both concentration and relaxation tasks were extracted and classified by using an LDA classifier. The classification accuracy of the AMM was calculated according to 10 × 10-fold cross validation. The *α* + *β* + *δ* + *θ* + *R* method was proposed by Liu et al. [7] to extract power features from multiple wavebands, including *α* band, *β* band, *δ* band, and *θ* band. The feature vector was defined as follows:

(7) $$\begin{array}{c} E=\left(\begin{array}{c} E_{\alpha }\\ E_{\beta }\\ E_{\theta }\\ E_{\delta }\\ R \end{array}\right)=\left(\begin{array}{c} \overset{13}{\underset{f=8}{\sum }}P_{f}\\ \overset{30}{\underset{f=14}{\sum }}P_{f}\\ \overset{7}{\underset{f=4}{\sum }}P_{f}\\ \overset{3}{\underset{f=0.5}{\sum }}P_{f}\\ \frac{E_{\alpha }}{E_{\beta }} \end{array}\right), \end{array}$$

where *E*_*α*_ is the power of brainwaves in *α* band and ∑_*f*=8_^13^ *P*_*f*_ denotes the sum of power spectrum between 8 Hz and 13 Hz. The *α* + *β* + *δ* + *θ* + *R* method then classified the feature vector *E* using a polynomial-kernel support vector machine (SVM) classifier. The accuracy rate of the *α* + *β* + *δ* + *θ* + *R* method was also calculated according to 10 × 10-fold cross validation.

## 3. Results and Discussion

### 3.1. Statistical Results of Raw EEG Data

**Figure 3:**
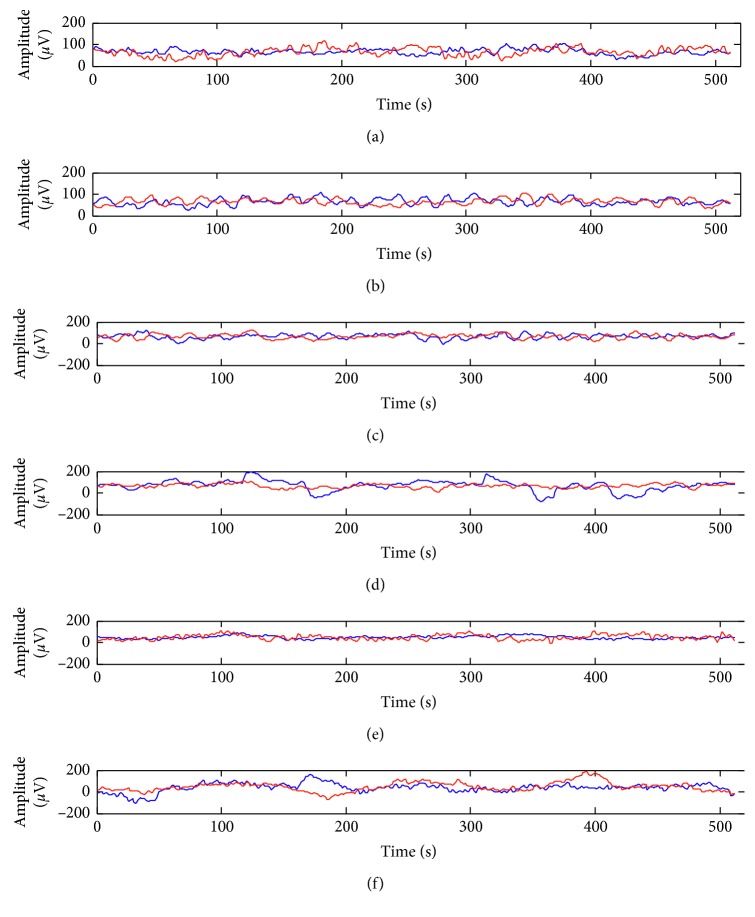
Average brainwaves in the concentration and relaxation tasks. The blue curves represent brainwaves of the concentration tasks, and the red curves represent those of the relaxation tasks.

The raw EEG data during the experiments were acquired and recorded using the Sichiray headset.  Figure 3 shows the average brainwaves acquired from the six subjects in the concentration and relaxation tasks. The blue and red curves represent the average brainwaves of the concentration and relaxation tasks, respectively. To compare the statistical characteristics of the raw EEG data, mean values, standard deviation, skewness, and kurtosis were computed for each epoch of raw data and were analyzed for the concentration and relaxation tasks, respectively.  Table 1 lists the statistical characteristics of the EEG data from six subjects. The results showed that the statistical characteristics were significantly different from individual to individual. This is caused by the low SNR of EEG signals and the inherent differences of individual's brain structures. Among the four statistical characteristics, the mean values showed no significant difference between the two tasks. However, the skewness values were higher in the relaxation task than those in the concentration task for all the subjects. However, these characteristics have rather large standard deviations, and it is difficult to separate these features between the concentration and relaxation tasks.

### 3.2. OCNM Features of Concentration and Relaxation

Table 2 lists the results of three parameters in constructing the OCNM for each subject. The optimization details are described in Section 2.3. *τ* is the delaying time and *m* is the embedding dimension, as in equation (1). *θ* is the threshold deciding the connectivity between two nodes, as in equation (3). The results showed significant difference in parameter values for varied individuals. As a result, these parameters should be optimized for a new user before the OCNM was applied to estimate his attention level. In our 10 × 10-fold cross validation evaluating the classification performance, 36 epochs of training data were used to estimate the optimized parameters for each subject.

**Figure 4:**
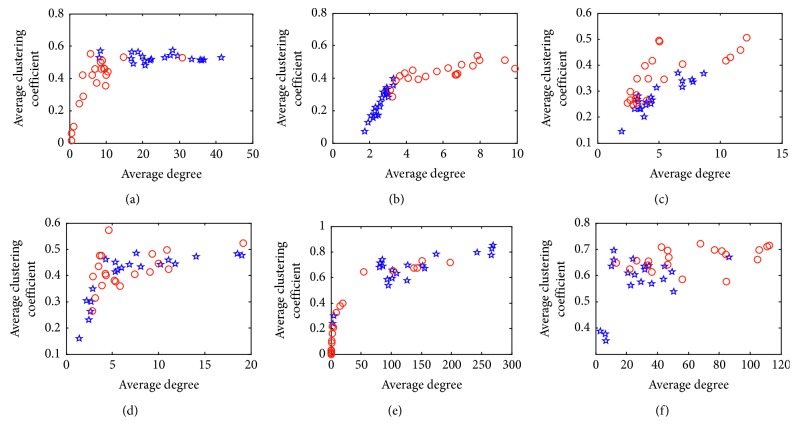
OCNM features of the concentration and relaxation trials for the six subjects.

Figure 4 shows the distribution of the OCNM features acquired in the concentration and relaxation tasks for the six subjects. The red circles denote features of the concentration trials, and the blue pentagons denote features of the relaxation trials. The results showed significant difference between the OCNM features of the concentration trials and the relaxation trials. An interesting fact is that for Subj1 and Subj5 (as in Figures 4(a) and 4(e)), the average degree and the average clustering coefficient are bigger in the concentration trials, while for Subj2, Subj3, and Subj6 (as in Figures 4(b), 4(c), and 4(f)), those features are bigger in the relaxation trials. This may be caused by the individual difference and requires detailed investigation in our future research. As shown in Figure 4(d), the OCNM features of two classes are difficult to be separated. As a result, Subj4 achieved the poorest classification performance among all the subjects.

**Figure 5:**
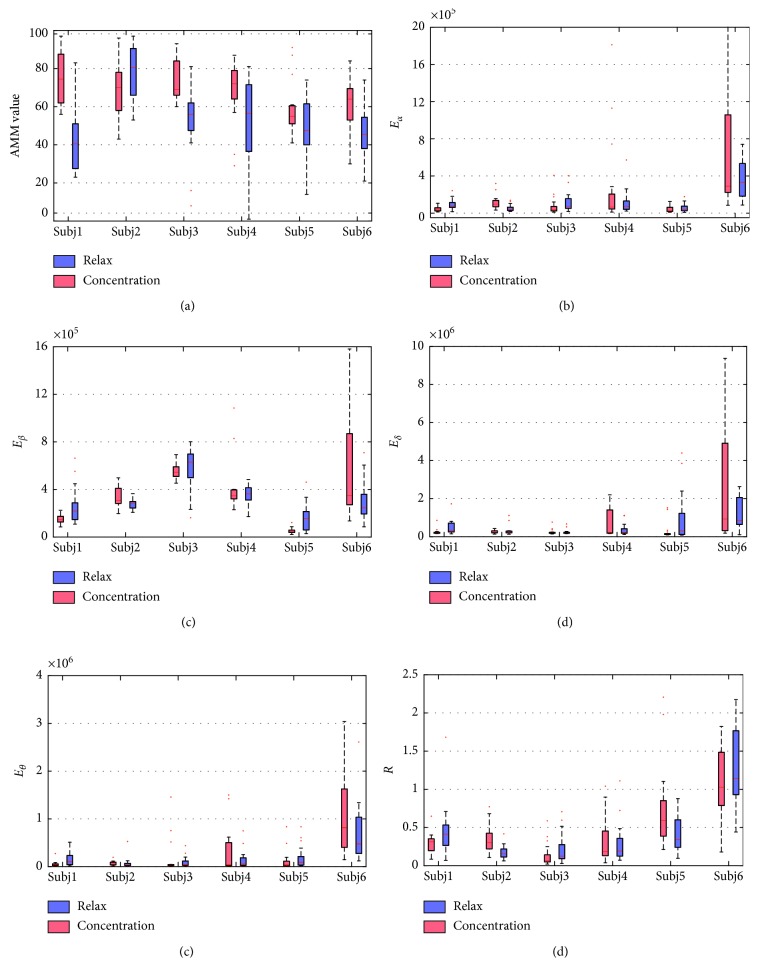
Statistical results of features extracted by the AMM and *α* + *β* + *δ* + *θ* + *R* methods.

Figure 5 shows the statistical results of features extracted by the AMM method and the *α* + *β* + *δ* + *θ* + *R* method. The red boxes denote the results of the concentration task, and the blue boxes denote the relaxation task.  Figure 5(a) shows the AMM values extracted from the six subjects, and Figures 5(b)–5(f) shows the features of the *α* + *β* + *δ* + *θ* + *R* method as in equation (7). These results reflected the individual variability of brainwave features. In our experiments, the classification parameters were trained for each subject, respectively.

### 3.3. Classification Performance of the Proposed OCNM

Table 3 lists the classification accuracy of the proposed OCNM for 6 subjects and compares its performance with the AMM and *α* + *β* + *δ* + *θ* + *R* methods. Please note that all the accuracy rates in Table 3 were calculated according to 10 × 10-fold cross validation. The results showed that the proposed OCNM delivered a higher average accuracy (80.67%) than the AMM (70.58%) and the *α* + *β* + *δ* + *θ* + *R* method (68.88%). Most of the subjects achieved the highest accuracy rate using OCNM, while for Subj4, AMM delivered a higher accuracy (69.00%) than OCNM (61.25%). An interesting fact for Subj4 is that all the methods delivered accuracy rates of below 70%. This may be caused by his bad execution of the experiments. Among the 6 subjects, Subj2 achieved the highest accuracy of 92.50% using OCNM, but his accuracy rate of AMM (58.75%) was the lowest. This result revealed the inherent difference of the methods' suitability for varied subjects.

**Figure 6:**
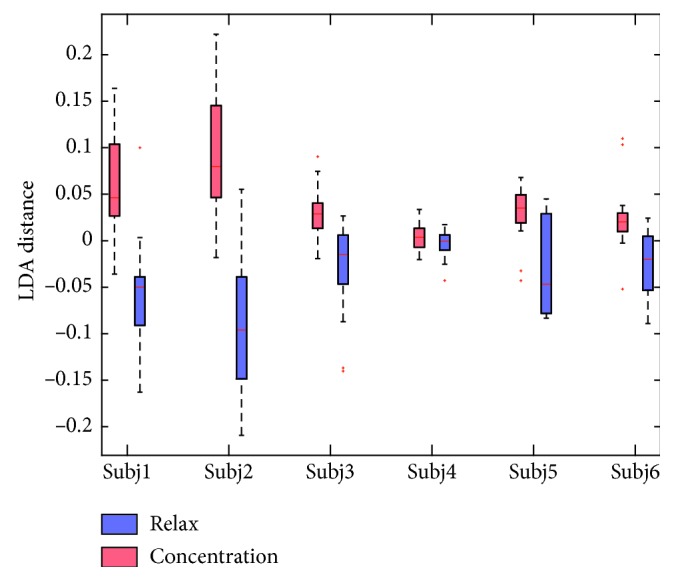
Statistical results of LDA distances for the six subjects.

In the offline analysis, the OCNM was used to classify the EEG signals into two brainwave patterns, i.e., concentration and relaxation. However, in a real-world neurofeedback system, it is necessary to measure more level of the attention for adjusting the strength of biofeedback. As in equation (6), the LDA classifier calculated the distance *d* of the OCNM feature vectors from the hyperplane and generated the classification results according to the sign of *d*.  Figure 6 shows the statistical results of the LDA distances. The *t*-tests were performed to compare the LDA distances between the concentration and the relaxation trials. For Subj4 who achieved an accuracy of 61.25% using the OCNM, the LDA distances of the concentration and relaxation trials are overlapped. The *t*-test also showed an insignificant difference (*p*=0.083) between these distances. For other subjects, the *t*-test showed significant differences (*p* < 0.05). Especially for Subj2 who achieved the highest accuracy of 92.5% using the OCNM, the significant level of the LDA distances are less than 0.01 between the concentration (*d* = 0.0909 ± 0.0641) and the relaxation (*d* = −0.0909 ± 0.0739) trials. This suggests that the LDA distance can potentially be used as a meter for measuring more levels of the human attention.

**Figure 7:**
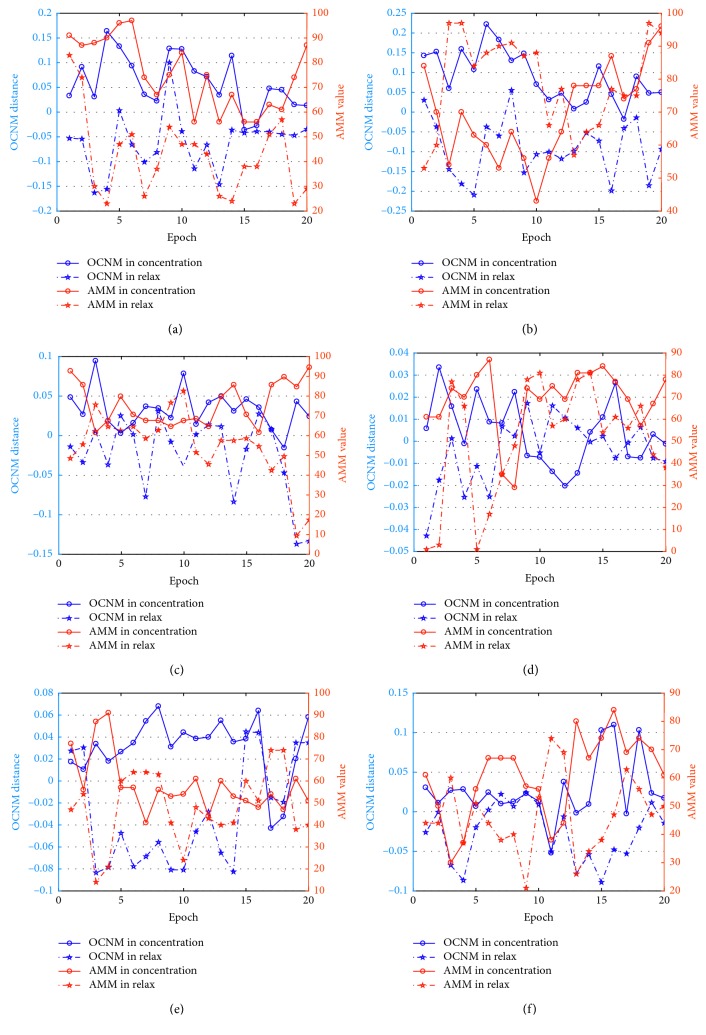
LDA distances of OCNM and AMM values obtained in each epoch of six subjects.

Figure 7 shows the LDA distances of OCNM and the AMM values of each subject obtained in all the epochs, consisting of 20 epochs of the concentration task and 20 epochs of the relaxation task. The horizontal axis indicates the epoch number for the recorded data, and the vertical axes represent the OCNM values and the AMM values, respectively. The red curves in the figures denote the OCNM results of each epoch, and the blue curves denote the AMM results. Among the curves of OCNM and AMM, the solid curves with circles denote the results acquired in the concentration task, and the dashed curves with pentagons denote the results acquired in the relaxation task. As shown in Figures 7(b) and 7(e), the red solid curves representing the concentration task were overlapped with the red dashed curves representing the relaxation task. As it is difficult to set a threshold to separate these features of different tasks, the AMM only achieved an average accuracy rate of 59.25% for Subj2 and Subj5. While for the blue curves denoting the OCNM results, it is easier to separate the blue solid curves and the blue dashed curves. As a result, the OCNM achieved an average accuracy rate of 86.25% for Subj2 and Subj5. For Subj4 who achieved poor accuracy rates using both methods, the red and blue curves in Figure 7(d) are overlapped for most of the epochs. The results indicate that the LDA distance of OCNM is more effective in representing subjects' attention levels.

## 4. Conclusions

In this paper, a novel OCNM method is proposed to improve the accuracy of measuring the attention level in the single-electrode neurofeedback system. The experimental results of six subjects showed that the OCNM achieved a higher accuracy rate (80.67%) than the AMM (70.58%) and the *α* + *β* + *δ* + *θ* + *R* method (68.88%). However, all these methods were only tested in the offline experiments. In an offline experiment, because of influence of classification output and real-time adjustment of the individual, the online classification performance usually differs from that in the offline experiments [20]. In future studies, we will improve the classification accuracy of OCNM in measuring more attention levels and report its online performance in a real-time neurofeedback system produced by Jiangsu Brain Medical Technology Co. Ltd., China.

## Figures and Tables

**Table 1:** Statistical characteristics (UV) of EEG data.

Subject	Concentration task				Relaxation task			
	Mean	Standard deviation	Skewness	Kurtosis	Mean	Standard deviation	Skewness	Kurtosis
Subj1	66.2184 ± 6.5575	51.5515 ± 17.6197	0.0092 ± 0.2459	2.9304 ± 0.5268	66.7563 ± 13.1456	87.8188 ± 28.1199	0.1698 ± 0.5107	3.8440 ± 0.7586
Subj2	65.7047 ± 6.0907	69.6273 ± 7.8738	0.0632 ± 0.1538	2.6919 ± 0.2462	65.3797 ± 4.3176	63.1947 ± 21.9904	−0.1455 ± 0.2179	2.9030 ± 0.7030
Subj3	66.6949 ± 2.2683	81.1608 ± 29.1632	−0.0489 ± 0.1872	2.8732 ± 1.1791	66.4806 ± 4.7196	79.5186 ± 14.8823	−0.0567 ± 0.2395	2.3520 ± 0.4298
Subj4	68.4021 ± 10.8351	207.1069 ± 157.6459	−0.0429 ± 0.3705	3.0295 ± 0.9266	65.2050 ± 4.9490	79.8901 ± 24.6297	0.1493 ± 0.4965	3.2224 ± 1.2867
Subj5	46.9497 ± 5.0841	53.1047 ± 38.3672	−0.0463 ± 0.7347	4.7057 ± 2.4036	48.5428 ± 14.0186	94.3458 ± 58.0666	0.1217 ± 0.5275	3.7828 ± 0.8419
Subj6	39.0700 ± 25.2965	205.6053 ± 110.5538	0.1608 ± 0.6567	3.6216 ± 1.5767	47.8521 ± 24.5879	160.6372 ± 35.8890	0.3500 ± 0.8969	4.8080 ± 2.2678

**Table 2:** Optimized OCNM parameters for six subjects.

Subject	*τ*	*m*	*θ*
Subj1	5	8	100
Subj2	6	9	90
Subj3	9	7	115
Subj4	7	9	110
Subj5	7	9	115
Subj6	5	7	200

**Table 3:** Offline classification accuracy rates (%) of six subjects.

Subject	AMM	*α* + *β* + *δ* + *θ* + *R*	Proposed OCNM
Subj1	84.75	77.75	**90.00**
Subj2	58.75	80.75	**92.50**
Subj3	80.25	66.50	**86.00**
Subj4	**69.00**	56.75	61.25
Subj5	59.75	74.25	**80.00**
Subj6	71.00	57.25	**74.25**
Average	70.58	68.88	**80.67**

## Data Availability

The data used to support the findings of this study are available from the corresponding author upon request.
